# Remarks on a Person Who Had Been Beheaded

**Published:** 1840

**Authors:** 


*Anatomico-Physiological Remarks on a Person who had been Beheaded.*—Some very curious and interesting remarks on a person who had been beheaded are recorded in *Miller's Archives* for 1838, by Professor Bischoff, of Heidelberg. We extract the following particulars from a translation by Dr. Bigger, published in our esteemed cotemporary, the *Dublin Journal of Medical Science*, (Sept., 1839.)

The individual upon whom the following observations were made was a robber and murderer, named Sebastian Zink, who was publicly beheaded on the 6th of July, 1838. Professor Bischoff and Dr. Heerman stationed themselves immediately under the scaffold, so that they could have the head and body of the decapitated man immediately under inspection, whilst Dr. Jolly remarked on the outside every thing which should occur, the greater part of which could also be seen and heard by his two colleagues.

"A few seconds," says Professor B., "after the blow with the sword had been struck, at thirty-six minutes and a half past nine, we received the head, which was hastily handed down in the bandage which had covered the face, without having been deranged by falling or receiving any shock. The bandage was immediately removed, in order to see the manner of separation from the body, the bleeding, and the impression of the features. The blow had been given most successfully, and the blade had passed anteriorly between the os hyoides and the larnyx, posteriorly very nearly between the fourth and fifth vertebræ, yet in such a manner that a portion of the left oblique process, and of the body of the fourth and a piece of the oblique process of the fifth were hewn through. The blood flowed slowly and continually. The expression of the countenance was of the most perfect tranquillity, not a trace of pain or distortion. The eyelids were a little sunk, the mouth closed, yet easily opened. The expression of the eyes was perfectly tranquil, neither staring, as mentioned by an earlier observer, nor yet dull and without lustre, but like those of a person who looks at something at a great distance. Dr. Heerman, who had visited and sat with the criminal for an hour on the preceding evening, could not discover any greater change in the features than the absence of his accustomed piercing, sharp glance, and the pupils being somewhat dilated.

"I approached my fingers, and then a brilliant needle close to the eyes, yet without touching either eyes or eyelids. This was repeated often without causing the slightest motion in either the eyes, eyelids, or other features.

Dr. Heerman now shouted his name in his left ear, and the word 'pardon,' under the conviction that this sound would produce an effect upon whatever consciousness might be present, as up to the last moment the criminal had been continually hoping for a reprieve. There was no trace of any motion. I now held to his nose a bottle of very strong smelling tincture of assafœtida, which preparation I preferred to ammonia, because I expected a purer action from it on the olfactory nerves, as it might be possible that the ammonia, by stimulating the nerves of the mucous membrane, should produce a motion of reflection. This experiment also failed. I then dropped upon his tongue a drop of the tincture of coloquintida; a feeble thrusting forward of the tongue and slight motion of the jaw ensued; both these motions were repeated three or four times, at short intervals, whilst in the other features no trace of motion was visible. In order to try, in a certain degree, how far the spirits of wine contained in the tincture could be regarded as cause of the motions, I placed a drop of pure spirits of wine on the tongue, which caused a similar motion of both tongue and jaw.

"All these experiments were finished within one minute after the fatal blow had been given. The question now arises, were those feeble motions of the tongue and jaw the consequence of sensation? if so, consciousness must have been present; or were they reflected motions dependent on irritation of the mucous membrane of the tongue, or from irritation of the severed spinal marrow, and therefore not caused by the tincture of coloquintida? To both of us the last of these suppositions appeared most probable. The tranquil condition of all the other muscles of the face appeared to contradict the idea that any unpleasant taste or flavor had caused these motions. The symptoms of the reflex functions, however much developed in the motions of chewing and swallowing, are much less affected by irritants applied to the tongue than by those applied to the mucous membrane of the palate, uvula and jaws. I must mention, also, that real irritation of the mucous membrane of the tongue with the point of a pin produced no motion. On the other hand, opening and shutting of the mouth are the motions which are most frequently to be seen in decapitated animals, and which are certainly dependent on the violent irritation of the spinal marrow. The tongue can move very little at the same time, on account of the attachment of the muscles which retract it having lost their fulcrum.

"Thus there does not appear to have been a single symptom present which permits us to suppose that consciousness was present; it appeared to have ceased instantaneously. It is right to remark that previous to the blow being given, he was not at all confused in mind, as were the ten criminals beheaded by the executioner Brand, as communicated by Heim. Zink, previous to execution, was in a perfectly careless mood, yet a little excited, and displayed the lamentable rudeness of his general demeanor; his mental powers were so little affected, that he said to the attendants of the executioner, as they were binding on the bandage on his eyes, an instant before he received the blow, 'Ye slaughter one like butchers!' During life this man seemed to possess very little nervous sensibility, the same was evidenced after death, in the experiments performed, so the evidence of those who have obtained contrary results is not by this instance much weakened. Yet I may say that negative results of an observation in the state of mind in which the greater number of observers must be at such a moment, carry with them more credence than positive ones, in which the excited fancy may play a prominent part, as from the relations of others, who during similar operations previously have beheld marvellous things. All the evidence brought forward with any accuracy to prove the continuance of consciousness, have consisted mainly in a few remarks on impressions being conveyed to the auditory apparatus in animals which had been irritated, which collectively cannot be considered as motions of reflexion, or as caused by irritation of the spinal marrow.

"I now proceeded to perform certain experiments from which I expected real motions of reflexion. I touched with a needle the eyelids and eyelashes as well as the conjunctiva; I irritated the mucous membranes of the nose, mouth and pharnyx, in the expectation of perceiving motion, but all in vain; all the muscles remained perfectly quiet, so that this was a proof not only of the rapid disappearance of consciousness, but also of that of the nervous irritability. Even piercing the severed part of the spinal cord with needles, and touching it with a pencil of kali causticum, produced no motions more in the head, whilst yet not more than from two to three minutes had passed from the period when it was severed from the body.

"We now turned our attention to the trunk, and whilst Dr. Heerman quickly tied the carotids, out of which the blood continued to flow with little jerks, in order to support the nervous and muscular irritability by detaining the blood, I sought, by piercing, scratching and pinching the skin on the soles of his feet, fingers and toes, to call forth some reflex motions; this proved vain also. Irritating the spinal cord produced twitchings in the pectoral muscles, and elevation of the arms.

"The corpse was now placed in a coffin and conveyed to the neighboring hospital, where every thing was ready for further experiments. It was now fifteen minutes past ten, consequently thirty-eight minutes and a half since the execution.

"We next introduced some electrical streams into the nerves. For this purpose Dr. Jolly had constructed a galvanometer, which by the immersion of a single plate of one-fourth inch square in pure water, produced an aberration of the magnetic needle to about 90°. I first sunk the platina terminations of the two conductors, one in the gray, the other in the white substance of the spinal marrow of the trunk. This process being repeated often, and the needles changed, not the slighest motion of the magnetic needle occurred; the introduction of the needles also caused no twitchings of the muscles. This experiment was tried on account of the assertion of Folchi, who stated that in a calf which had just been decapitated, and on which a similar experiment had been tried, there was an aberration of the needle abont 6° west, on every new introduction of the needles, (Foriep's Notizen, No. 950,) which experiment had often before been tried without any effect on decapitated dogs. Dr. Heerman now exposed the median nerve in the upper arm, and I plunged the two needles into the trunk of the nerve, at the distance of about one inch from one another, and endeavored, by mechanical irritation of the spinal marrow to produce twitchings in the arm; none occurred, nor were there any changes in the magnetic needle. I now applied one pole of a powerful galvanic battery of sixty pair of four inch plates, capable of giving me a tolerably strong shock, producing sparks, and decomposing water, upon the spinal marrow, the other on the hand. There arose evident yet slight twitchings in some of the muscles of the fore and upper arm, viz: in the supinator longus, extensor carpi, ulnaris longus, and in the internal head of the triceps, but no motion in the magnetic needle. It also remained perfectly unmoved when I applied the second pole upon the trunk of the nerve, the same was the case when I applied both poles to the trunk of the nerve, so that the chain was perfect, although in the latter instance twitchings occurred in the muscles mentioned above. Some inference may be drawn for the hypothetical electrical streams in the nerves, at least that the nerves are exceedingly good conductors of electricity, and much better than the metals, or otherwise the magnetic needle should have been affected in the last case, had it been the better conductor. Mattevic, in the Bibl. Univers. de Geneve, August, 1834, has communicated a similar experiment with like result. On the contrary, J. Muller has, in the Archiv. fur Anatomie, Phisiologie, &c., declared that all parts of the body, and even a drop of water act in the same manner. Without wishing to raise a doubt against this observation, we may say that from it cannot be deduced that the nerves and perhaps other moist organized parts are not good conductors. The want of reaction in the galvanometer, when the circle is completed, by any of these parts, will admit of the general conclusions being drawn either that they are very good or very bad conductors of electricity. The experiment of Muller admits only the one explanation, that the moist organic structures are very bad conductors, for he only employed a weak source of electricity, a single pair of moistened plates, and the circle was not completed, and thence there was no aberration of the magnetic needle. On the contrary, in our case the occurrence of reaction, the contraction of the muscles showed that the circle was completed. Should any one object that nerves are bad conductors, and that the results we obtained were from our using a very powerful source of electricity, his own argument might be used against him, for the more powerful the electricity, the more likely would it be to pursue the metallic conductor if it were the best. The non-reaction of the galvanometer appears to have been caused by the very great conducting susceptibilities of the nerves (or perhaps of the other animal tissues,) for electricity: I do not wish to draw as a conclusion that electricity is the active agent of the nervous system, a circumstance which for many other reasons I think to be improbable. I have repeated also the experiments of Varvasseaux and Berardi, as well as those of David, and in no instance has there been any change of position remarked in the magnetic needle when I sink the platina conductor of the galvanometer into the nerves of a living animal, and by means of mechanical irritation caused motions and twitches, actions of nervous irritability. This is accounted for, as we said before, from the nerves being better conductors than the metals.

"Mechanical irritation of the median nerve, pricking, pinching, and even cutting it across, produced no effects, whilst galvanism still produced twitchings.

"I next opened the chest and abdomen, whilst Dr. Heerman tried some experiments on the irritability of the iris. It was now fifty minutes past ten, and the iris showed no disposition to contract, not even when the pole of the battery was applied directly to it, the cornea having been removed, yet the battery was so strong that it immediately caused decomposition of the fluids of the eye, and a development of gas.

"In the chest and abdomen there were no spontaneous movements to be seen; also when the galvanic pole was applied consecutively to the phrenic and vagus nerves, to the stomach and intestinal canal, on the ureters, gall bladder, and the cystic duct, no contractions occurred. The right auricle of the heart alone continued to move after this, for an hour and a quarter, the same occurred in the muscles of voluntary motion, but was scarcely perceptible after an hour and fifty minutes. As the experiments on the phrenic and vagus nerves were made at ten minutes past eleven, just one hour and thirty-three minutes and a half from the execution, even then the twitchings in the voluntary muscles were very weak, in comparison with those mentioned by other observers in other cases, which confirms the remark which I made already with regard to the head, that the nervous faculty and capability of irritation were not strongly developed in this man.

"Whilst those observations were making, I was seeking the thoracic duct, which I found, with a good deal of trouble, on account of the body being very fat and the vessel containing only a muddy, grayish fluid; a portion of this fluid being removed, coagulated as usual, but it formed a very small coagulum around the bit of stick which was moved in it. Under the microscope the little round bodies did not appear numerous in the fluid, but were nearly of the same size as the molecules in the blood, many of which might have entered along with the chyle whilst we were obtaining it, but there is sufficient to distinguish these two sets of bodies from one another.

"I take advantage of this opportunity to communicate my observations on the chyle molecules, which I had often examined in dogs. In the white chyle of the thoracic duct of young dogs, after being fed, I have always seen two kinds, the first exhibits an innumerable quantity of exceedingly small, little bodies (kornchen) or particles, which are only to be seen when the chyle is permitted to run on plates of glass, when their appearance is exactly like that of sand put in motion. The microscope must be very powerful to show this. The second kind of little bodies are fewer in number, much larger, and the greater part of them of the size of the molecules in the blood, but there is no kernel and no envelope to be seen in or on them, besides they have not the yellowish appearance which, when powerfully magnified, even a single blood molecule exhibits ; they are unchangeable in water and in active acid. When the chyle is agitated they sink to the bottom, and they form a part of the coagulum. The rest of the fluid remains white, where they are in very small number, and where they could not be the cause of the intense white color. This color is, I think, attributable to the other set of innumerable little molecules, which I take to be fat. Still I am opposed to the opinion of J. Muller, that the white color of the chyle is principally attributable to fat; for if the chyle be treated with æther, in a glass tube, often shaken, and fresh æther still added, (not in a watch glass, where the experiment cannot succeed,) it loses nearly all its color, and appears of a light opal. This remnant of opacity cannot be caused by the larger molecules, for they were dissolved just as the blood molecules are by the æther, a process which I saw going on; but it seemed to me that this opacity arose from coagulated albumen. I believe that the evidence of Tiedeman, Gmelin, and J. Muller are alike, all considering the prevalence of the white color in the chyle as proceeding from very minutely divided particles of fat, but that these are not the genuine chyle molecules which exist independent of them. This view is strengthened by this, that in the pinkish contents of the duct in dogs which had fasted long, those very fine particles were not so evident, whilst the other chyle molecules were even as numerous, nay, relatively more numerous than usual.

"After having collected the chyle, I observed the mucous membranes of the trachea and larynx, and had the pleasure of seeing myself, and being able to show to those present, the ciliary motions. These motions were not discoverable in the œsophagus, not even in that part which covers the posterior wall of the cricoid cartilage.

"I next examined the urethra, vesiculæ seminales, vas deferens, and epididymis. In the urethra it was evident that an ejaculation of semen had taken place, as in the case given in Valentine's Repertorium, I. p. 277 ; this arises, naturally, from the violent contraction of all the muscles at the moment of decapitation. There was found in the urethra many large, yellowish coagula, like coagulated lymph, and a whitish fluid which contained mucous seminal animalculæ alive, but not so numerous as those which I have seen in the seminal fluid of other animals. The same kind of coagula, and the same animalculæ were seen in the vesicuæ seminales, which were of small size. The animalculæ were found in the whole course of the vas deferens, alive, but not in such great numbers. I thonght I could see them also in the seminal canals of the testis, but as they were few in number, and not living, I do not wish to assert this as a positive fact. The contents of these parts contained also many other kinds of particles, but none of such determined forms as those which Valentine describes. It is probable they were particles of epithelium. The fluid which exuded, on a section of the prostate being made, was clear and pellucid, but I could not discover in it any elementary bodies, except some blood molecules.

" Much pains were taken to examine the brain carefully; when the skull-cap was removed, all the veins were found to be full of air, and air had penetrated between the pia mater and arachnoid, causing a very peculiar appearance on the surface of the hemispheres. Could this air have entered at the severed part of the spinal cord? This is difficult to credit, for in the brain as well as in the spinal cord, the pia mater and the arachnoid lay very close together, and no fluid intervened, the escape of which might permit the entrance of air. Through rupture of the vessels? This I am not willing to allow either. And by this my view is strengthened, that the formation and course of the arachnoid is not yet perfectly discovered. The examination of the brain was not satisfactory. Particular attention was paid to the spleen: in it I found the little white bodies described by Malpighi, which I have seen in the four classes of vertebrated animals, but which are hard to discover clearly in man. I believe that the function of the spleen is to produce them, and I cannot see any reason why Muller should suppose that those he has seen in certain ruminating animals, are of a different nature from those which have been found in the spleens of other animals, or from those which I believe to exist in the healthy spleens of all men. That these are hard, and tolerably large in some, whilst in others they are soft, small, and easily dissipated, is no reason that they should be considered essentially different. I found, also, in these bodies, universally, as well as in the spleen of this subject, those little round bodies or balls which J. Muller has described. With a cataract needle it is easy to isolate as many of these little bodies from the spleen as may be necessary for microscopical observation. I find that these are exactly similar in appearance, size, relations to water and acetic acid, to those of the chyle; therefore is it not possible that these may originate in the spleen? It is true that the corpuscules of which the parenchyma of the liver consists, are, as Muller remarked, very like those in the corpusculi Malpighiani, yet similar bodies are to be found in many places, and still the parts organized from them are very different from one another.

"From attention to this point, I think shortly, much information wiil be obtained with regard to the spleen, and the formation of the corpuscules of the blood, although as yet no change has been seen in the formation of blood after extirpation of the spleen. It is questionable whether sufficiently accurate examination has been made of the blood globules in those animals which have been deprived of their spleens.

"Lastly, I examined the stomach and the villi of the intestinal canal. In the former the mucous membrane of the fundus was strikingly softened; it was now 4 o'clock, and the day very warm, the contents of the stomach smelt very sour, from wine and sallad. The villi of the small intestines were of pyramidal form, formed of little bodies united together, and I could not discover any epithelium formation on them after they had been gently washed in water.

"There was no diseased appearance in the whole body, except an adhesion of the lung to the walls of the thorax, occasioned by an old gunshot wound in all probability; three grains of large shot were found under the pleura costalis, unchanged in appearance, and surrounded by a quantity of cellular tissue.

"The ventricle of the heart was very strong and muscular, and compared with the rest of the muscular structures, might be called hypertrophied.

"After six hours busily and uninterruptedly employed with the body of this unfortunate man, though aided by my friends I still feel that many observations might have been made in a more accurate manner. My hope is, that what has been done may lead to further observation on the same subject."

*Amer. Jour. Med. Sci.*

